# The Institutional Library

**Published:** 1913-12-06

**Authors:** 


### THE INSTITUTIONAL LIBRARY.

A Guide to the Mental Deficiency Act, 1913. By John and Samuel Wormald. (London: P. S. King and Son. Pp. 145. Price 5s. net.)

For once in a way the reference of a great national question to a Royal Commission has not been equivalent to shelving reforms and shutting the Governmental eyes to remediable evils. It is true that the recommendations of the Royal Commission on the Feeble-Minded had been formulated some few years before legislation was able to incorporate them; but it is distinctly something unusual to find any good at all coming out of a Royal Commission, and also an encouragement for the future. There are (estimated to be) at this moment 150,000 persons in the kingdom who are not certifiably insane, yet are so feeble in mind as to be a nuisance to the community, a burden to their friends, and a danger to themselves. Unprotected, they become criminals, prostitutes, and the like; and they reproduce their kind with amazing facility. To take care of these, to classify them, and treat them according to the degrees of their defect, and generally to make the most of such intelligence as they possess, is the object of the Mental Deficiency Act. This Act is not very lengthy, as Acts go; nor is it obscure in its wording. But it sets up a new machinery which is sure to work a little stiffly at first. To ease the bearings and lubricate the machine is the object of this excellent introduction to the Act; and in our judgment it is admirably designed to achieve those objects.

The British Red Cross in the Balkans. Published under the auspices of the British Red Cross Society. (Cassell and Co. 1913. Price 1s. net.)

This book consists of a brief introduction summarising the work done by the British Red Cross Society in its attempts to mitigate the horrors of the late war in the Balkans, and of more than a hundred selected photographs showing incidents in the experiences of those whom the Society sent out. The modest space of three pages, to which the letterpress is confined, shows very clearly what a great work of humanity the Red Cross Missions accomplished; and forms a most excellent justification of the British Society's existence and a demonstration of how well it applies the funds entrusted to it. The photographs are, however, the *raison d'être* of the volume. They are extremely good, being doubtless the cream of many collections; and some of them would gain high marks at a photographic exhibition for "composition" and other attributes of artistic merit. We applaud the idea of advertising the British Red Cross Society by these striking examples of the work which it does.

### A Hospital Novel.

A White Passion. By A. B. Teetgen. (London: Wells Gardner, Darton and Co., Ltd. Price 6s.)

In this book by Miss Teetgen the story is told of the building, starting, opening, and all but financial disaster of a pioneer cottage hospital on the Canadian prairie in the Far West. It is truly a hospital novel, in the sense that the institution, with its interests and adventures, is the true hero of the tale, though the man who did the work and triumphed over all the unromantic discouragements and difficulties which beset any new social undertaking is Dr. George Liston, a dogged prairie practitioner, who sees how futile is one man's medical work largely in a scattered district where visits are paid by mileage, and where time is spent between the patients rather than with them. Committee-men especially should appreciate the story, for committee work, squabbles, achievements, grievances, and public spirit form the background of the tale, the realism of which in these respects deserves praise for its observation. We ourselves are too much hardened novel readers to appreciate the love motive which is introduced. It hardly helps the interest, and is too slight in itself to grip the reader. The novel, however, has a double value; it represents pioneer hospital work in the Colonies, and should be a comfort and inspiration to all interested in starting cottage hospitals, first-aid classes, or district nursing at home. It tells what difficulties organisers must expect, and points to tenacity and tact as the best means of surmounting them. Moreover, more skill than appears on the surface is required to make such instructive drudgery as meetings, interviews, and anxieties readable, as Miss Teetgen has certainly done.

### Smallpox Outbreak.

The Administrative Control of Smallpox: How to Prevent or Stop an Outbreak. By W. McC. Wanklyn, B.A., D.P.H. (London: Longmans, Green and Co. 1913. Price 3s. 6d.)

Vaccination having been allowed to fall from its high estate by reason of the facility with which exemption can now be obtained, it is comforting to know that there are some people alive to the fact that every year the danger of an epidemic of smallpox is increasing. When it will come no one seems able to prophesy, in spite of the cyclical prevalence of smallpox. Only two things are certain: susceptible material is increasing, and infection is continually coming in. This little book follows as a natural corollary to Dr. Wanklyn's other recently published volume on "How to Diagnose Smallpox." The subject of the control of this disease is, of course, of first importance to medical officers of health, and any day the public health authorities may be called upon to put forth strenuous efforts at a moment's notice. Dr. Wanklyn in this companion volume has presented his subject in a conversational manner. He deals fully with the principal administrative details which require to be put into practice in order to cut short an outbreak. The book was drafted originally for students reading for the Diploma of Public Health, but it is not unlikely that it will find its way on to the bookshelves of many medical officers of health. On the question of vaccination the author naturally takes up a firm attitude, but adds a sage piece of advice to the young medical officer of health —that is, never to be drawn into a dispute or discussion on the "vaccination question," except when giving advice to the authority who retains his services.

Lippincott's New Medical Dictionary: A Vocabulary of the Terms used in Medicine, Dentistry, Veterinary Medicine, and the Allied Sciences. By Henry W. Cattell, A.M., M.D. Illustrated. Third Edition. (Philadelphia and London: J. B. Lippincott Company. 1913. Price 21s. net.)

A Dictionary such as the one under notice offers something more than the mere meaning of a strange word; a certain amount of collateral information of a descriptive or encyclopædic character is also included, and this naturally increases its utility, especially to laymen. At the present time even a well-read medical man will find he needs to look up some new word or expression quite frequently, for the specialists are only too fond of coining fresh ones. A glance at the list of only some of the new words incorporated in this new edition will bear this out, and incidentally impress on the reader the extent of his own ignorance. Lippincott's dictionary has already established itself with considerable firmness, and its features are generally familiar. Immense pains have been taken to ensure correctness, and much ingenuity has been displayed in the saving of space by conciseness and meticulous arrangement. In spite of its 1,100 pages the book is of a handy size; it is, moreover, clearly printed, has a thumb index of the alphabet, and is very conveniently bound in serviceable limp covers.

### Hobbies Made Easy.

First Steps in Collecting. By Grace Vallois. (T. Werner Laurie, Ltd.)

It is difficult to decide whether a book of this sort, which might have been called "Collecting Made Easy," really appeals to the hobby instinct, or, as at least is equally probable, to the modern lip-service to "culture" and the well-informed mind. Oscar Wilde, in one of his most inspired moments, said: "A well-informed mind (we quote from memory) is a dreadful product. It is like a bric-à-brac shop, all monsters and dust, with everything priced above its proper value." Yet it is true to say that the well-informed mind, though exactly what this fine observer described it, is the desirable thing to most of us. Hence, among other things, "chatty" books about collecting. Madame Vallois, keen, equipped with the collector's knowledge, and with, it appears, sufficient means to gratify it handsomely, makes the time-honoured apology for the "chatty" and informing guide: "My books," she says, "are not learned—there are plenty of those—but aim at enticing readers towards more robust efforts." Thus in three parts the book describes and illustrates the more famous examples of old furniture; old pottery and glass; the tea-table of the eighteenth century; and the flotsam and jetsam of old lumber rooms. From a literary point of view, the "chat" interferes somewhat unpleasantly with the information; but this last, though designedly brief, does give the chief facts of importance concerning the fine and the fake in each case. The authoress evidently knows her subject, and likes to communicate her knowledge, but with her interest so great as it appears to be, we wonder that she has sacrificed her passion to the supposed needs of the tyro in the self-sacrificing way that this sort of writing demands. Certainly one cannot stop at her book. She has achieved her aim in making the reader desire more, but with the *arrière-pensée* that more might have been given if the "chat" (it is her word) had been curtailed.

Through a Glass Darkly. By Trevor Blakemore. (Gay and Hancock. Price 6s.)

The novel about artists is still popular, though we may prophesy that the novel about doctors will rival it in public estimation before long; as soon, that is, as a capable story-teller is found who knows the world of medicine and has no particular bias in favour of any section of it. Stephen Andrew has come, but greater work than he has yet done is required for a medical "Trilby." In the meantime, the novel about artists still holds the field. There is no need to dwell on the curious and rather cumbersome machinery, with its suggestion of the occult, which forms the vague framework of this book. Evidently the author himself does not care much about it, and his volume must stand or fall by its description of the art world of London and Paris. The author, there is no doubt, has seen or read much about this world, but is not a subtle observer, and his characters are therefore not fascinating figures. They are too much like the people we all know, using that phrase as it is always used concerning those who have not excited us enough to regard them as other than such loosely defined types as a rich Philistine, a serious-minded girl, a bounder, and the like. Still, there are some neat and humorous things. The criticism of the Puritan temperament as "more occupied with the rejection of evil than with the absorption of good" is an example of the first. The astonishingly naïve appraisement of Renée's "beautiful nature" as one which "sacrificed her very moods" to the man she lived with is an example of the second. If we could be sure that Mr. Blakemore were only expressing the views of Renée's particular lover, and not his own deepest views of what is most beautiful in the feminine kind, we should welcome him as a fine and subtle critic, so exquisitely does this appraisement represent the point of view of respectable men. This is what the word "womanly" means to them. As one of the best relaxations for a man in one profession is to read about the *vie intime* of another, we can cordially recommend this novel to all art-interested members of the institutional and medical world.

The Management of Children in Health and Disease. By Mrs. Usher. (London: J. and A. Churchill. 1913. Price 1s. net.)

The authoress of this book has, we gather, had no actual medical or nursing training; but is an intelligent mother, intensely interested in the welfare of her own children, and anxious to pass on the knowledge and experience she has acquired to others. Her good intentions cannot be doubted, and the greater part of her advice is thoroughly sound and judicious. There are, however, what a schoolmaster would call "howlers" here and there, which seem to have escaped the notice of the medical man who read the manuscript. On the whole we feel obliged to say that there are better books of this size and price and aim. So many have been published on this subject lately that only the very exceptionally excellent can expect to make elbow room for themselves in the crowd.

### Latin for M.R.C.P. Examinees.

Latin Self-taught. By John Topham, Barrister-at-Law. Pp. 144. Cr. 8vo. In red cloth, 1s. 6d.; blue wrapper, 1s. (London: E. Marlborough and Co. 1913.)

To those who are familiar with Marlborough's "Self-taught" Series of manuals for the rapid acquisition of a foreign tongue, it may come as somewhat of a shock to find that Latin has been included in such a series. Intended for tourists and others who seek short cuts to linguistic facility, these books have successfully catered for them. But until Latin has been resurrected and popularised sufficiently to oust Esperanto (a consummation devoutly to be desired, in the writer's estimation), it is not likely that such a text-book as this will be taken up by academies or even polytechnics. Nevertheless, this book will be an excellent means of reawakening the dormant knowledge of Latin of many professional men, and for candidates for the M.R.C.P. examination it will prove a very present help in time of trouble. To tourists and antiquarians it is conceivable that this volume will be of assistance in the interpretation of inscriptions and records. As a condensed grammar the book is useful for revision, but it is hardly to be recommended for the purpose of learning the language. The so-called reformed pronunciation has been adopted throughout, and this is given phonetically in the vocabularies, which include those giving medical, dispensing, and chemical terms.

